# Evaluation of a Blended Physical Activity Intervention for Older Adults: Mixed Methods Study

**DOI:** 10.2196/16380

**Published:** 2020-07-23

**Authors:** Sumit Mehra, Jantine van den Helder, Bart Visser, Raoul H H Engelbert, Peter J M Weijs, Ben J A Kröse

**Affiliations:** 1 Applied Psychology Faculty of Applied Social Sciences and Law Amsterdam University of Applied Sciences Amsterdam Netherlands; 2 CREATE-IT Applied Research Faculty of Digital Media and Creative Industries Amsterdam University of Applied Sciences Amsterdam Netherlands; 3 Informatics Institute Faculty of Science University of Amsterdam Amsterdam Netherlands; 4 Center of Expertise Urban Vitality Faculty of Health Amsterdam University of Applied Sciences Amsterdam Netherlands; 5 Center of Expertise Urban Vitality Faculty of Sports and Nutrition Amsterdam University of Applied Sciences Amsterdam Netherlands; 6 Department of Rehabilitation Amsterdam University Medical Centers University of Amsterdam Amsterdam Netherlands; 7 Department of Nutrition and Dietetics Amsterdam University Medical Centers VU University Amsterdam Netherlands; 8 Amsterdam Public Health Research Institute Amsterdam University Medical Centers VU University Amsterdam Netherlands

**Keywords:** frail elderly, aged, activities of daily living, exercise, health behavior, telemedicine, mobile devices, tablet computers, usability testing, evaluation

## Abstract

**Background:**

Physical activity can prolong the ability of older adults to live independently. Home-based exercises can help achieve the recommended physical activity levels. A blended intervention was developed to support older adults in performing home-based exercises. A tablet and a personal coach were provided to facilitate the self-regulation of exercise behavior.

**Objective:**

In line with the Medical Research Council framework, this study aimed to carry out process evaluation of a blended intervention. The objectives were (1) to assess the long-term usability of the tablet adopted in the blended intervention and (2) to explore how the tablet, in conjunction with a personal coach, supported older adults in performing home-based exercises.

**Methods:**

The process evaluation was conducted with a mixed-methods approach. At baseline, older adults participating in the blended intervention were asked to fill out a questionnaire about their general experience with information and communication technology (ICT) devices and rate their own skill level. After 6 months, participants filled out the Usefulness, Satisfaction, and Ease of use (USE) questionnaire to assess the usefulness, satisfaction, and ease of use of the tablet. With a random selection of participants, in-depth interviews were held to explore how the tablet and coach supported the self-regulation. The interviews were double coded and analyzed with the directed content analysis method.

**Results:**

At baseline, 29% (65/224) of participants who started the intervention (mean age 72 years) filled out the ICT survey and 36% (37/103) of participants who used the tablet for 6 months (mean age 71 years) filled out the USE questionnaire. Furthermore, with 17% (18/103) of participants (mean age 73 years), follow-up interviews were held. The results of the baseline questionnaire showed that the large majority of participants already had experience with a tablet, used it regularly, and reported being skillful in operating ICT devices. After 6 months of use, the participants rated the usefulness, satisfaction, and ease of use of the tablet on average as 3.8, 4.2, and 4.1, respectively, on a 5-point scale. The analysis of the interviews showed that the participants felt that the tablet supported action planning, behavior execution, and self-monitoring. On the other hand, especially during the first few months, the personal coach added value during the goal setting, behavior execution, and evaluation phases of self-regulation.

**Conclusions:**

The results of the process evaluation showed that older adults who participated in the study were positive about the blended intervention that was designed to support them in performing home-based exercises. Participants reported that the tablet helped them to perform the exercises better, more frequently, and safely. It supported them in various phases of self-regulation. The availability of a personal coach was nevertheless crucial. To support physical activity in older adults, a blended approach is promising.

## Introduction

### Background

As people age, they face a decline in daily functioning and mobility [1,2]. Physical activity can delay the onset and slow the decline associated with aging [3,4]. Older adults who exercise on a regular basis can prevent impairments and remain self-reliant for a longer period of time [5,6]. Accordingly, various community centers around the world offer senior citizens the opportunity to participate in group-based exercise classes under the guidance of an instructor [7-9]. For instance, in the Netherlands, over 400,000 older adults participate in the weekly activities of “More Exercise for Seniors” (“*Meer Bewegen voor Ouderen*,” which is abbreviated as MBvO in Dutch). Despite the popularity of this program, its effects on physical health are limited. A previous study has shown that older adults who participate once a week in the exercise classes, do not achieve a higher health-related quality of life or an increased ability to perform daily tasks [10]. In order to capitalize on the health benefits of physical activity, the frequency, intensity, and duration of exercises have to be sufficient [9,11].

Older adults can increase the level of physical activity by doing exercises at home, either as an independent program or in conjunction with group-based classes [12-16]. The latter approach combines the motivational aspects of exercising along with peers with the flexibility of a home-based exercise program that is tailored to individual needs. However, in the absence of an instructor, older adults may have adherence and safety concerns about home-based exercises [17]. The use of mobile technology (mobile health [mHealth]) can help overcome these issues by providing detailed instructions, offering tailored programs, and tracking progress [18-23] for individuals, including older adults [24,25].

### Development of a Blended Intervention

In order to enhance community-based exercise programs like MBvO, a blended intervention was developed as part of the MOTO-B (Motivating Technology for Older Adults’ Behavior) and VITAMIN (VITal AMsterdam older adults IN the city) research projects. The aim of the intervention was to support older adults in performing home-based exercises. In line with the self-determination theory [26,27], the intervention was conceived to increase competence and stimulate the autonomy of older adults, but at the same time, to maintain relatedness with peers [17]. The intervention consisted of a home-based exercise program that was supported by a tablet and a personal coach, and could be followed alongside community-based exercise programs or other sport activities.

### Objective

According to the UK Medical Research Council (MRC), complex interventions need to be evaluated systematically [28]. Three different types of evaluations can be distinguished as follows: (1) assessing the feasibility, (2) assessing the effectiveness, and (3) understanding the underlying change process.

First, prior to assessing effectiveness, feasibility should be investigated thoroughly. For the blended intervention described here, a previous usability study that was conducted in a laboratory showed that older adults (age ranging from 69 to 99 years) who used the app for the first time during a 45-minute session could operate it without any relevant problems [29], suggesting that the blended intervention is feasible. However, a more thorough evaluation is needed to account for the long-term use in a real-world setting. The usability of mHealth apps is often not tested sufficiently, thereby limiting their effectiveness [30,31].

Second, the effectiveness of a complex intervention can be assessed with randomized controlled trials (RCTs). To assess the effectiveness of the blended intervention in terms of health outcomes, a trial study is currently ongoing and will be reported in the future elsewhere [32].

Third, an explorative process evaluation can provide insights into the underlying change process. By exploring the mechanisms of action, a process evaluation is a valuable extension of effectiveness studies. The aim of this study was to conduct such an evaluation. The objectives were as follows: (1) to assess the long-term usability of the tablet in a real-world setting and (2) to explore how the tablet, in conjunction with a personal coach, supported older adults in performing home-based exercises.

## Methods

### Intervention

**Figure 1:**
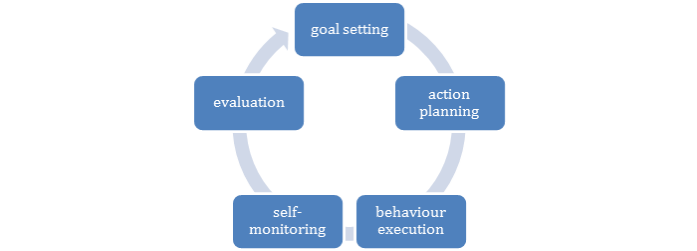
Behavior change through self-regulation.

The intervention consisted of two components to support older adults in performing exercises at home. The first component was the tablet containing a custom-developed app that was designed to ensure behavior change by facilitating self-regulation. Self-regulation is the process of consciously guiding one’s own behavior in order to achieve goals. In particular, behavior change techniques that support goal setting, action planning, behavior execution, self-monitoring, and evaluation appear to be important for the self-regulation of behavior [33-35]. Figure 1 presents a schematic representation. The app supported self-regulation by allowing older adults to set goals, tailor a weekly schedule to their individual needs, and watch video instructions. It also tracked their progress and facilitated remote guidance by a personal coach. An elaborate description of the app and its theoretical underpinning have been presented previously [36].

The second component of the intervention was counselling. Each participant was appointed a personal coach. The coach paid house visits, helped the participants to get acquainted with the tablet, and counselled, either remotely or face-to-face, the participants in setting up and following the tailored exercise schedule. The coaches were third- and fourth-year physical therapy bachelor students. Prior to taking on their responsibilities, the coaches received a 2-week training on functional exercises, good clinical practice, and e-coaching by faculty staff members. Furthermore, during their 6-month internship, they received weekly supervision from faculty staff members. When the responsibilities of a coach ended at the end of a teaching semester, ongoing cases were transferred to a new coach. As a result, participants received in sequence counselling by two personal coaches during a 6-month period. The complete details of the intervention have been reported previously [32].

### Study Design and Participants

An RCT was conducted to assess the effectiveness of the blended intervention in terms of health outcomes. Older adults were recruited from the surroundings of Amsterdam, the Netherlands, through postal mailing and local community-based centers offering weekly exercise programs. Applicants were included in the trial if they met the following criteria: (1) age 55 years or older, (2) ability to understand the Dutch language, and (3) absence of specific cognitive or physical impairments. The protocol that describes the RCT has been published, including detailed methods, inclusion criteria, measurement procedures, and interventions [32].

To increase the fidelity of the trial, an additional nested mixed-methods study was set up (described in this paper) by administering questionnaires to the participants who received a tablet and coaching, as well as conducting follow-up interviews among a random selection of those participants [37].

### Measurements

Before commencing the intervention, at baseline, the trial participants filled out a three-item questionnaire about their general experience with information and communication technology (ICT) devices, such as computers, smartphones, and tablets. To assess the long-term usability of a tablet (objective 1), after 6 months, participants who received a tablet were asked to fill out a usability questionnaire that was based on the Usefulness, Satisfaction, and Ease of use (USE) questionnaire [38]. The first part of the questionnaire consists of 23 items (Likert) that measure the following three components of usability: usefulness, satisfaction, and ease of use. Each item consists of a statement with the following five response options: strongly disagree, disagree, neither agree or disagree, agree, and strongly agree. The second part of the questionnaire contains three general questions about tablet use in the past 6 months, whether participants would recommend the tablet to friends, and an item participants could use for general remarks. All questionnaires were administered by paper and pencil in the Dutch language.

Furthermore, to explore how the tablet, in conjunction with the coach, supported the self-regulation of exercise behavior (objective 2), 18 participants were randomly selected for an in-depth interview. The interview questions were previously piloted among two participants. The interviews were conducted in a home setting, were held in Dutch, and lasted for about 45 minutes. All interviews were recorded.

### Analysis

The questionnaires were processed with double-entry verification. For usefulness, satisfaction, and ease of use, separate mean scores were calculated. The mean scores could range from 1 (very low) to 5 (very high). The interviews were transcribed verbatim and subsequently double coded by two researchers. The directed content analysis method was used to explore how the participant’s experience related to the five key constructs of self-regulation that the intervention was based upon (goal setting, action planning, behavior execution, self-monitoring, and evaluation). Directed content analysis was deemed more appropriate than conventional content analysis, because of the focus on existing theoretical constructs [39-41]. To minimize differences in interpretation, first calibration sessions were held. Subsequently, both researchers coded all the transcripts independently with the key constructs of self-regulation and then compared the results. Differences were resolved via discussion. In the rare case no interrater consensus was reached, the first author settled the dispute.

## Results

### Questionnaire About ICT Experience and Skills

In total, 224 older adults with a mean age of 72 years (SD 7 years; 71% female) participated in the RCT at baseline. The questionnaire about the prior use of ICT devices and self-reported skill level was filled out by 29% (65/224) of the participants, of which 72% (47/65) were female. Their mean age was 71 years (SD 5.8 years). The tablet was one of the most popular devices among the participants. A large majority of the participants used this device several times a week. See Table 1 for the results.

Most participants rated themselves as somewhat skilled with ICT devices. Specifically, 6% (4/65) of the participants rated themselves as very unskilled, 2% (1/65) as unskilled, 38% (25/65) as somewhat skilled, 40% (26/65) as skilled, and 6% (4/65) as very skilled.

**Table 1:** Prior use of information and communication technology devices (N=65).

Device^a^	Use, n (%)				
	Never	Rarely^b^	Sometimes^c^	Regularly^d^	Often^e^
Personal computer	17 (26%)	1 (2%)	3 (5%)	8 (12%)	24 (37%)
Laptop	17 (26%)	3 (5%)	3 (5%)	7 (11%)	25 (39%)
Tablet	16 (25%)	2 (3%)	3 (5%)	3 (5%)	35 (59%)
Smartphone	13 (20%)	0 (0%)	2 (3%)	3 (5%)	41 (63%)
Mobile phone^f^	25 (39%)	1 (2%)	2 (3%)	5 (8%)	14 (22%)

^a^The values of individual items are less as some items were skipped by the participants.

^b^Once a year or less.

^c^Few times a year.

^d^Few times a month.

^e^Few times a week.

^f^Device without touchscreen.

### Usability Questionnaire

The usability questionnaire was filled out by 36% (37/103) of the participants who had used the tablet for 6 months, of which 60% (22/37) were female. The mean age was 71 years (SD 5.1 years). The questionnaire had excellent internal consistency with Cronbach α of .89. The internal consistencies for the subscales were as follows: usefulness, .82; ease of use, .89; and satisfaction, .71.

Participants indicated that they found the tablet very useful (item 1) and it helped them to perform their exercises better (item 3) and safely (item 4). They were, however, neutral about how this affected their daily lives (item 6 and item 7). Overall, they were satisfied with the tablet and found it easy to use. Table 2 presents the results of the USE items. Finally, 68% (25/37) of the participants indicated that they would recommend the tablet to friends. Participants who indicated that they would not recommend the tablet provided varying reasons like “it didn’t work properly,” “I don’t need it to be active,” or “it’s too noncommittal.”

**Table 2:** Scores of usefulness, ease of use, and satisfaction items (5-point Likert scale) (N=37).

Questionnaire item^a^		Score^b^, mean (SD)
**Usefulness**		3.8 (0.6)
	1. The tablet is useful.	4.5 (0.6)
	2. With the tablet, I can follow an individual exercise program that suits me.	4.0 (0.7)
	3. The tablet helps me to perform my exercises better.	4.2 (0.7)
	4. With the tablet, I can perform exercises safely.	3.9 (0.9)
	5. The tablet helps me to perform exercises more often.	3.8 (1.1)
	6. Since using the tablet, I have a more active life.	2.9 (1.0)
	7. The tablet supports my daily activities.	3.0 (1.1)
	8. The tablet has everything I need to be physically active.	3.6 (1.1)
	9. The information about the exercises is understandable.	4.4 (0.6)
**Ease of use**		4.2 (0.6)
	10. I learned to use the tablet quickly.	3.9 (1.1)
	11. I easily remember how to use the tablet.	4.3 (0.9)
	12. I am capable of using the tablet.	4.2 (0.8)
	13. I can use the tablet without any help.	4.3 (0.7)
	14. I understand how the tablet operates.	4.3 (0.7)
	15. I can easily find what I am looking for on the tablet.	4.2 (0.9)
	16. The tablet is easy to use.	4.4 (0.7)
	17. Using the tablet is effortless.	4.4 (0.5)
**Satisfaction**		4.1 (0.6)
	18. I am satisfied with the tablet.	4.2 (0.7)
	19. The tablet is pleasant to use.	4.1 (0.8)
	20. The tablet is fun to use.	4.0 (0.7)
	21. I am going to keep on using the tablet.	4.0 (1.1)
**Miscellaneous**		
	22. Family and friends believe I should use the tablet.	1.9 (0.9)
	23. The trainer/coach believes that I should use the tablet.	3.3 (1.3)

^a^Translated from Dutch.

^b^The minimum score is 1, and the maximum score is 5.

### Interviews

In total, 17% (18/103) of the participants who used the tablet for 6 months were approached for a follow-up interview. One participant declined without giving a specific reason. The interviews were conducted with the remaining 17 participants, of which 53% (9/17) were female. Their mean age was 73 years (SD 7.0 years). The results of the interviews are described below according to the following five phases of self-regulation: goal setting, action planning, behavior execution, self-monitoring, and evaluation.

#### Goal Setting

Goal setting involves the process of determining the objective a person aspires. Setting goals was the departure point of the blended intervention. The tablet was designed to support the participants by letting them rank a set of daily activities and subsequently formulating their goals. It was also the main topic of the first two meetings with the coach.

The participants’ goals varied greatly (ie, from decreasing backache to improving balance). For some participants, the goal was not to improve physical health but to maintain it. Occasionally, participants formulated the goals with only the tablet, but most participants first consulted with the coach to explore related issues and translate top-level goals to specific and challenging, but realistic and measurable, goals. The attention the coach paid to the individual situation of the participant was appreciated. For instance, participants commented as follows:

…then we looked what is useful for me, what will help me to improve?... well, this was decided in consultation.

Participant #14

I believe it is important that the coach kept in mind: what does this person want to achieve?

Participant #6

well… they asked me about everything… which problems do I face?

Participant #1

In summary, the tablet, in conjunction with the coach, supported the participants in setting goals. The sensitivity of the coach for the personal circumstances was valued by the participants.

#### Action Planning

Action planning involves the process of making a plan regarding how the goals will be achieved. After determining the goals, participants could draw up a personal exercise schedule on the tablet. They could select functional exercises that would increase balance, strength, flexibility, and endurance. Each exercise was available in three variations that differed in difficulty.

The choice of different exercises was valued. For instance, participants commented as follows:

... then you always can choose your own exercises. I think it is great you have a lot of choice.

Participant #4

That’s good. Then I can adjust it entirely to my own needs.

Participant #6

Some participants commented that customizing the exercise schedule was not easy to do, either because of technical limitations of the tablet or because of limited knowledge about the benefits of each exercise. In those cases, the coach was available to help. For instance, participants commented as follows:

…well, which exercise should you choose? … that I could do this together with my coach was very effective.

Participant #4

with his help I had in no time an entire exercise program.

Participant #3

The weekly overview of planned exercises helped the participants to be physically active. The majority of participants exercised daily. They commented that this was due to the intervention as follows:

I am chaotic and have no discipline, this helped me a lot!

Participant #17

I do the exercises every day at home. I did not do that before.

Participant #13

now I am consistently doing exercises, every day. Actually, because of this [tablet].

Participant #10

When asked about the underlying reason for this, they mentioned different aspects. Several participants indicated that the tablet provided them structure to build a routine. For many, this was doing the exercises at a fixed time of day, generally in the morning. Participants commented as follows:

before taking a shower and getting dressed, first those exercises. A fixed structure, that helped.

Participant #2

…well, that rhythm is a good feeling.

Participant #15

Others commented that the exercises were more integrated in their daily activities as follows:

…sometimes I also do the exercises as I go; then I walk step by step back into the living room after a visit to the bathroom.

Participant #11

…I do the exercises in between times. I stand on one leg when I am brushing my teeth for instance. Well, I kind of integrate it.

Participant #14

Besides providing structure, some participants mentioned that the tablet also acted as a cue to action as follows:

...when I sit down and see it [tablet] I think ‘ah, a reminder!

Participant #16

In summary, participants felt that the blended intervention supported them in action planning. It provided them with structure to develop a routine. Several participants indicated that it helped them to do exercises daily, a frequency they previously did not achieve. They valued the possibility to personalize the exercise schedule to their own needs. The help of the coach was essential for some participants.

#### Behavior Execution

Behavior execution involves performing the actual behavior that should lead to achieving the goals. The tablet was designed to support this by various features like giving an overview of today’s exercises, providing background information about each exercise along with video demonstrations, and providing a countdown timer or the ability to modify each exercise with three parameters (duration, number of repetitions, and intensity level).

Participants found the daily overview of exercises to be useful. It provided them in a brief glance which exercise had to be performed today and with what duration, repetition, and intensity level. The countdown timer was used especially in the beginning when participants had to familiarize themselves with the exercise routine. The same applied for the video demonstrations. It helped them to see how the exercises could be performed correctly. For instance, participants remarked as follows:

…but I did need it [video demonstration] to do it [the exercise] in the correct manner.

Participant #6

…that was nice, I could perform the exercises better this way.

Participant #7

Additionally, attention to safety was valued, with the following statements:

the exercises are safe. Well, at least a lot safer than riding a bike. Biking is dangerous.

Participant #12

…yes, attention was paid to this [safety]. That you had to hold on to something, when you stand on one leg, for instance.

Participant #6

One of the participants stressed the benefit of using a tablet for the instructions as follows:

I can write it down, but it’s nice to have visual image of what is meant…. instructions written down are always subject to different interpretations. I think, as it has been done now, is very instructive.

Participant #4

Nevertheless, numerous participants mentioned that the additional instructions of the coach were also valuable as follows:

I also asked the coach, “am I doing it right?” He said “yes, that’s right” or “you have to do it like this and that”.

Participant #11

…that was nice. Sometimes he would demonstrate the exercise, or I would demonstrate it and ask him if I was doing it correct.

Participant #6

The coach also helped participants modify exercises if they were struggling with limitations or wanted more of a challenge. The latter was often needed. Many participants stressed that the exercises were too easy, despite the possibility to increase the difficulty level with the tablet. Apparently, this was not sufficient for numerous participants. An illustrative remark was as follows:

…yes, I can say that I wished they were a bit more challenging.

Participant #10

Some felt very strongly about this. For instance, a participant remarked as follows:

Look, I believe these exercises are meant for people who are in a retirement home and, more or less, don’t do anything the entire day.

Participant #15

Two participants indicated that they stopped doing the exercises because of this reason. Others found creative ways, together with their coach, to increase the intensity level, for instance, by increasing the repetitions, skipping breaks, or adding weight. For instance, a participant commented as follows:

…such as the exercise with shopping bags… I added dumb-bells to it, now it’s really challenging.

Participant #10

Finally, as participants developed a routine, they relied less on the tablet and on the coach for performing the exercises. Some participants kept on having the tablet in sight during the performance of the exercises, while others merely glanced at which exercises had to performed today and then executed them without the tablet. Watching the video demonstrations or using the countdown timer was not needed anymore. In some cases, participants even did all the exercises by heart, and one participant mentioned the following:

... I can do the exercises when I am at work, in between times. I just count the exercises myself.

Participant #8

When asked about the necessity of a coach, most of the participants felt that after 2 or 3 months, the coach’s help was not needed anymore.

In summary, the video demonstrations and countdown timer helped the participants to perform the exercises safely and correctly, especially during the early stages of the intervention. The coach played an important role in adapting the exercises to meet the capacity of the participants, as many of them sought a bigger challenge. In time, the participants developed a routine and performed the exercises more autonomously.

#### Self-Monitoring

Self-monitoring involves the process of keeping track of one’s progress. The tablet was designed to support this by letting users tick off exercises that had been completed. In a weekly overview, users could see which exercises had been done and which had not been done. Additionally, a progress bar indicated how many exercises still had to be done today and for the current week. Furthermore, the coach could remotely monitor the progress of the participants.

The moment at which participants ticked off exercises varied. Some did this directly after completing the exercises, whereas others did it at the end of the day. The majority of participants felt that keeping track in this manner gave them insights into their own behavior and was motivating. For instance, one participant made the following statement:

…for me it’s very easy…it gives insight and lets me follow what I have done.

Participant #9

Remarkably, various participants expressed that the mere action of ticking off exercises was not only easy but also rewarding. It left them with a feeling of accomplishment. For instance, one participant made the following statement:

…look, in the end you want to finish off your list.

Participant #8

However, the progress bar, which indicated how many exercises were completed, was hardly used. Many participants did not seem to have noticed this feature, indicating a usability issue. A couple of participants also expressed the desire for more advance features to investigate their progress, like graphs and tables.

Several participants mentioned that remote monitoring by the coach was an important factor for them to keep doing the exercises. For instance, some participants remarked as follows:

…the tablet motivates me, but…I must say. I think this is also because…that it is being monitored.

Participant #7

…I think it helps… there is someone keeping an eye on you.

Participant #14

you are participating in study, you want to show that you are cooperating.

Participant #10

On the other hand, other participants indicated that this was not the case for them. They would keep doing the exercises if there was no coach involved.

In summary, keeping track of progress with the tablet was easy and motivating. Ticking off completed exercises was experienced as rewarding and gave participants insights into their progress. For some participants, the fact that they were remotely being monitored was motivating, while for others, the social presence of a coach was not important.

#### Evaluation

Evaluation involves the process of reflecting on the effort and the progress that has been made in relation to the goals that were set out to be achieved. First, the tablet was designed to support the evaluation process by letting participants rate each exercise on three aspects (effort, complexity, and enjoyment). Second, either via video calls on the tablet or with face-to-face meetings, the participants had the opportunity to reflect on the progress together with their personal coach.

The ability to rate exercises with the tablet was superfluous according to several participants. The need to evaluate each exercise after completion seemed tedious. One participant made the following statement:

Well, look. This bothers me. I think ‘come on guys. Everything is so easy and simple. For me there is no difference in it [the effort, complexity or enjoyment of the various exercises].

Participant #2

Some participants suggested that it would have been better if they could rate exercises on a weekly basis instead of on a daily basis or only when they felt the need to do so. In contrast, the participants were more positive about the evaluation with the coach. They felt that it helped them to identify issues. Several participants mentioned, however, that toward the end of the 6-month intervention, the coaching was not needed anymore.

Finally, some participants reported that they experienced an improvement in vitality. They found themselves to be in a better shape than before and attributed this to the blended intervention. One participant made the following comment:

…yes, I now really get up without any backache, although this was previously the case. The pain returns in the evening when I am tired, but in the morning it’s different. That is a huge benefit.

Participant #5

Others did not notice an improvement, despite performing exercises, but also expressed more modest expectations. Maintaining their health status was more important than achieving progress for them, as indicated by the following remarks:

Do I notice an improvement in the gym? No. But if I don’t do my exercises for a week or two…then I can notice the difference.

Participant #6

I notice, I am 85, that I am declining… my goal is to stay steady.

Participant #4

Another participant mentioned the following:

…when you are 18 you can expect to keep on getting better, but for me, after one year I am even more old again… Can I perform some exercises that I couldn’t do before? Sure. In that sense there is progress. But it isn’t so that I am going to keep on improving.

Participant #16

When asked if they would like to keep the tablet for exercising, the vast majority of participants expressed the wish to do so, regardless of whether they notice an improvement.

In summary, the blended intervention supported participants in evaluating their progress. Specifically, the conversations with the coach were responsible for this. Overall, the participants evaluated the blended intervention to be useful. Some felt that their health improved, but others did not have this feeling. Nevertheless, almost all participants indicated that they wanted to continue their exercise routine with support of the tablet.

## Discussion

### The Value of the Blended Approach

The objectives of this study were to assess the usability of the tablet and how it supported older adults in performing home-based exercises, in conjunction with a personal coach. A previous usability study showed that first-time users (age ranging from 69 to 99 years) could successfully complete various predefined tasks on the tablet during a 45-minute session in a laboratory [29]. This study extends those findings by showing that the tablet can be not only successfully operated in a standardized setting for a short period of time, but also useful, satisfying, and easy to use within the context of exercising at home during daily life for an extensive period of time. The participants indicated that the tablet allowed them to follow a tailored exercise program that suited them. It also helped them to perform the exercises more often, better, and safely. From the perspective of older adults, it can be concluded that the use of the tablet successfully supported them in their exercise behavior.

The interviews revealed a more detailed view on the underlying processes. The tablet was useful in developing an exercise routine. The tablet supported the participants in action planning and behavior execution by providing them with a tailored schedule that gave structure and video instructions demonstrating the appropriate behavior. Furthermore, ticking off exercises as a simple form of self-monitoring appeared to be motivating. On the other hand, the interviews revealed that the personal coach played an essential role. The interactive and social nature of coaching was especially useful during the self-regulation phases of goal setting and evaluation. The ability to interact with users in this manner is yet to be achieved by a virtual coach or avatar [24,42,43]. In addition, although the tablet allowed users to tailor the exercise program to their own needs, the exercises in the app did not sufficiently match the needs of the participants. The expertise of the coach was crucial for adapting the exercises to accommodate preferences. Finally, the presence of the coach in the form of remote monitoring was motivating for some participants. These findings are in line with other research showing that physical activity interventions incorporating access to a remote expert for advice and social support tend to be effective [34,44,45]. This study indicates that the coach might, in particular, be beneficial during the initial period, when participants familiarize themselves with the intervention and develop a routine.

### Improvements

Although the participants were overall positive about the blended intervention, the evaluation also revealed several possibilities to improve the intervention. First, fit older adults should be able to add more challenging exercises to their schedule. Taking into account the preferences of some older adults, adding support for outdoor activities to the tablet would be enriching. For instance, a map with walking trails in the vicinity could stimulate older adults in achieving daily physical activity. Second, the tablet should offer more detailed reports of user progress (eg, graphs that display long-term trends). Some participants requested such a feature. Third, the extent participants relied on a coach varied from person to person. Owing to the protocol of the RCT, coaches contacted the participants with a fixed frequency. When the intervention is implemented in practice, the intensity of counselling should be tuned to the preferences of individuals. Presumably, some older adults will extensively make use of counseling, while others will merely limit it to initial support.

### Study Limitations

The aim of the blended intervention was to support older adults in performing home-based exercises. Questionnaires as well as interviews showed that older adults felt that the intervention accomplished this. However, an underlying assumption of the intervention was that regularly performing exercises would support older adults in their daily activities and lead to an active lifestyle and an increase in their vitality. The interviews showed mixed results on this topic, and no support was found for these assumptions from the questionnaires. More challenging exercises or a more comprehensive approach for the vitality of older adults might be needed for such secondary effects; however, the effectiveness of such strategies is also debatable [46-50].

The results of the questionnaires have to be interpreted with caution though. All older adults who participated in the clinical trial were given at the start of the trial a questionnaire about their prior experience with ICT devices. Only 65 of the 224 trial participants completed this baseline questionnaire. Furthermore, the 6-month trial had a 18% drop-out rate. Among the remaining 103 older adults who were part of the group that received a tablet, only 37 filled out the USE questionnaire about the usability of the tablet. The usability results may therefore be biased. Perhaps only participants who had a positive experience with the tablet filled out the usability questionnaire. We do not, however, think this is plausible. First, the baseline questionnaire had a high rate of nonresponses. This fact cannot be explained by a negative experience with tablet use in the blended intervention. The participants were yet to embark on the intervention when filling out the baseline questionnaire. The high nonresponse rate for both the questionnaires might have been caused by the numerous tests that were administered by the researchers as part of the larger clinical trial [32]. The testing procedure, including body measurements, took half a day. This might have led to fatigue, causing participants to skip questionnaires. Second, the positive evaluation based on the questionnaire is in line with the results from the interviews. Although the sample size of the interviews was small, it was not susceptible to selection bias. The interviews were based on random selection of participants. Only one participant declined to be interviewed. Therefore, the sample that was drawn for the interviews can be considered to be representative of the older adults participating in the intervention. The previous usability study among first-time users and the questionnaires and interviews of this study all point in the same general direction of a favorable evaluation.

The extent to which the findings can be generalized to older adults in general is a different issue. The baseline questionnaire showed that prior use of tablets was high among the participants. Studies have shown that among older adults, tablets are easier to operate than smartphones or personal computers owing to the large touchscreen [51-53]. This was one of the reasons to choose a tablet as the delivery device for the blended intervention [36]. The usability of tablets can also explain the increasing popularity of tablets among older adults. In the United States, tablet ownership among adults aged 65 years or older rose from 1% in 2010 to 32% in 2016 [54]. In the Netherlands, a similar trend has taken place, where tablet ownership among those aged 65 to 75 years grew from 28% in 2012 to 60% in 2016 [55]. In this light, the prior use of tablets among the participants of this study is representative of the larger population. Nevertheless, perhaps only older adults with a positive attitude about ICT in general or a tablet in particular signed up to participate in the blended intervention. More research is needed to assess how a wider range of older adults will experience a physical activity intervention that incorporates the use of tablets.

### Conclusion

A mixed-methods process evaluation showed that older adults are positive about a blended intervention designed to support them in performing home-based exercises. Participants rated the adoption of a tablet as useful, satisfying, and easy. They indicated that it helped them to perform exercises better, more frequently, and safely. It supported them in various phases of self-regulation. The interactions with a personal coach strengthened this by offering deeper reflection and more fine-grained tailoring during the earlier stages of the intervention. A blended approach appears to be a promising strategy for delivering physical activity interventions in older adults.

## Abbreviations

MBvO: Meer Bewegen voor Ouderen; More Exercise for Seniors, a Dutch community-based physical activity program for adults aged 55 and above
mHealth: mobile health
MOTO-B: Motivating Technology for Older Adults’ Behavior
MRC: Medical Research Council
RCT: randomized controlled trial
USE: Usefulness, Satisfaction, and Ease of use
VITAMIN: VITal AMsterdam older adults IN the city
